# Voicing narratives of structural violence in interpersonal firearm violence research and prevention in the United States

**DOI:** 10.3389/fpubh.2023.1143278

**Published:** 2023-06-02

**Authors:** Shani A.L. Buggs, Julia J. Lund, Nicole Kravitz-Wirtz

**Affiliations:** Violence Prevention Research Program, Department of Emergency Medicine, University of California, Davis School of Medicine, Sacramento, CA, United States

**Keywords:** community violence, structural violence, firearm, prevention, equity, narrative

## Abstract

Violence is defined as “the intentional use of physical force or power, threatened or actual, against oneself, another person, or against a group or community, that either results in or has a high likelihood of resulting in injury, death, psychological harm, maldevelopment, or deprivation.” Encompassed in this definition are multiple, interrelated forms of violence, including interpersonal firearm death and injury, but also the systems, policies, and practices enacted by those with power to advantage some groups while depriving others of meaningful opportunities for meeting their basic needs—known as “structural violence”. Yet dominant violence prevention narratives too often ignore or deemphasize the deeply intertwined threads of structural violence with other forms of violence, leading to policies and practices that are frequently insufficient, and often harmful, for reducing interpersonal firearm violence and building community safety, particularly in minoritized and structurally marginalized communities. We highlight ways in which limited scrutiny of structural violence, the omission of its defining characteristics—power and deprivation—from functional characterizations and frameworks of interpersonal firearm violence, and the inadequate distribution of power and resources to those most impacted by violence to self-determine narratives of and solutions to interpersonal firearm violence grossly impacts how interpersonal firearm violence is collectively conceived, discussed, and addressed. Expanding dominant narratives of interpersonal firearm violence, guided by the wisdom and determination of those most impacted, such that the goal of prevention and intervention efforts is not merely the absence of violence but rather the creation of a community safety and health ecosystem is essential to meet this critical moment in firearm violence research and prevention.

## Introduction

Violence, particularly violence involving a firearm, is among the most challenging and devastating public health problems in the United States (US). More than 45,000 Americans lost their lives to firearm violence in 2020, the highest absolute number of gun deaths ever recorded, with an alarming increase in deaths by community gun violence (1, 2), broadly considered to be firearm violence between non-intimately related parties, generally occurring outside the home (3). The firearm homicide rate in the US increased by 35% between 2019 and 2020, reaching its highest level since 1994; preliminary data suggest an even further increase in 2021 (2). And yet deaths due to gun violence capture only a fraction of the human toll of this problem. While reliable data on the number of people who are shot but survive is not widely available (4), it is estimated that non-fatal firearm injuries outnumber firearm fatalities by more than 2 to 1 (5). Countless more individuals and families live in neighborhoods plagued by the collective grief and anticipatory trauma (6) caused by secondary and pervasive experiences of day-to-day incidents of community gun violence (7–10).

Violence is defined by the World Health Organization (WHO) as “the intentional use of physical force or *power*, threatened or actual, against oneself, another person, or against a group or community, that either results in or has a high likelihood of resulting in injury, death, psychological harm, maldevelopment, or *deprivation*” (emphasis added) (3). As translational researchers committed to equitable, anti-racist approaches to the study and practice of firearm violence prevention, we assert that dominant violence prevention narratives fail to engage the voices of those most impacted and, as such, too often ignore or deemphasize, at least implicitly and at times intentionally (11), what are arguably the most fundamental component parts of this definition—namely, power and deprivation. In so doing, the concept of structural violence (12)—systems, policies, and practices enacted by those with power to advantage some groups while depriving others of meaningful opportunities for meeting basic needs critical to safety and health—has been allowed to fade from mainstream public conversation, rather than bridging the work of interpersonal firearm violence prevention with deliberate action to challenge the systems of power that create conditions that lead to increased risk of community gun violence in the first place.

We currently have a unique window of opportunity—at a time when the COVID-19 pandemic has laid bare the persistence and consequences of interrelated forms of oppression and inequity; amid nationwide organizing for racial justice in response to police violence; and as elected officials across levels of government have made unprecedented commitments to invest in community violence intervention and prevention—to build on this “anti-inequity” (13) reckoning to re-narrate the structural roots of community firearm violence and reimagine and remake its solutions. Yet the language and stories that are heard, elevated, believed, and acted upon will continue to be bounded by the racialized framing of individual-level drivers of criminality, dangerousness, and blameworthiness, and thus produce inadequate and at times harmful, mostly enforcement-based approaches to public safety, unless we are intentional in naming the deeply intertwined threads of structural and community (gun) violence.

At the same time, narrating community firearm violence through this structural lens cannot be a solely deficit-based discourse. Rather, and significantly, the language of structure and agency, and by extension, of violence and peacemaking, must exist in tandem. Structural violence does not happen in a vacuum but instead is perpetually challenged, disrupted, and debated within minoritized and structurally marginalized communities at the same time it is being produced (14). Equitable, anti-racist approaches to community gun violence prevention thus require not only naming and addressing structural violence and community violence as co-constituted and historically contingent, but also uplifting the actions, attitudes, and stories of healers and peacemakers in communities most impacted by this violence, understanding their individual and collective energies as a reimagining and remaking of the structures intended to constrain them and as key leverage points for change. To this end, our call for structural explanations and solutions to community violence, while being rooted in an understanding of the racialized institutional forces which concentrate inequitable conditions associated with violence and safety, rejects the idea that any community is inherently or inevitably violent and instead centers the many strengths of community.

## Intertwined threads of structural and community violence

Community gun violence, as already noted, has been defined as violence that occurs between non-intimate parties, primarily in public spaces and involving the use of a firearm. While this definition allows for distinction from some other forms of gun violence, such as firearm suicide or intimate partner firearm violence, it is nonetheless an overly broad category, encompassing forms of gun violence involving rape or sexual assault by strangers and mass violence in schools or workplaces, in addition to the community firearm violence that is the focus of this piece, which often involves interpersonal or intergroup conflict between people who know each other and which is concentrated in racially and economically segregated communities. This lack of specificity in the definition of community firearm violence, particularly given the complex nature of this problem, invites, perhaps even necessitates, the application of racialized schemas and mental heuristics that inevitably push the mainstream narrative toward individual-level (and mostly deficit-based) risk factors and behavior change interventions that are seen as more proximal to interpersonal violence, further pathologizing environmentally-responsive survival strategies and reinforcing status quo approaches based on reductionist explanations that do not threaten the very real structural and institutional arrangements that govern the pervasiveness of interpersonal firearm violence among certain populations and in certain places.

Community gun violence, like so many other harmful health exposures, is not evenly distributed across the US. While public, mass-casualty shootings in malls, concert venues, clubs, and schools may capture our society’s collective horror, attention, and calls for action, these incidents accounted for less than 3% of all interpersonal gun deaths in 2020 (15). An overwhelming majority of the remaining firearm deaths, in 2020 as well as in the years both preceding and since, are concentrated in communities and among people that have been affected by and subjected to historical and present-day structural racism and inequity. Firearm homicide has been the number one cause of death for Black males between the ages of 15 and 34 for over 30 years; it is the second-leading cause of death for Latino males and Black females ages 15–24 (16). More than half of Black youth and nearly half of Latinx youth in large cities in the US live within 1,300 meters (the approximate radius of a census tract) of a past-year firearm homicide occurrence, with 1 in 4 Black youth and 1 in 5 Latinx youth living near 3 or more incidents in the past year; the comparable rates for white youth are 17% near any incident and less than 1% near 3 or more incidents (9). Research has also shown that Black and Latinx youth in middle-to-high income households are nearly twice as likely as white youth in low-income households to live or attend school near a deadly firearm violence incident (8).

Racialized structures in the US have guided the laws and practices governing how and which communities do and do not receive systematic social and economic support, which profoundly influences the concentration of community firearm violence. In particular, place-based discriminatory lending and residential mobility policies and practices, including but not limited to forced residential segregation, redlining, urban renewal projects of the 20th century, racialized restrictive covenants, and the underappraisal of home values and economic capital in predominantly Black neighborhoods, have led to systematic disinvestment in minoritized communities over generations, with underinvested neighborhoods experiencing significantly higher rates of firearm injury compared to those that have benefited from more consistent social and economic investment (17–21). Consequently, neighborhood disadvantage has been shown to play an even greater role than household-level economic disadvantage in inequities in community gun violence exposure; researchers have found that for all racial and ethnic groups, the difference in the probability of exposure to a past-year firearm homicide between youth in low vs. high poverty households is approximately 5–10 percentage points, while the difference between youth residing in low vs. high disadvantage neighborhoods is approximately 50 percentage points (9). Furthermore, though recent research has importantly begun to examine how historical practices such as redlining have influenced present-day interpersonal firearm violence trends, it must be noted that contemporary actions that perpetuate racialized health, economic, and safety disparities, such as mass incarceration (22, 23), state-sanctioned violence (24), economic policies (25), inequities in quality food availability and security (26, 27), and even environmental pollution (28), require similar scrutiny for systematically harming the physical and mental wellbeing of marginalized populations and communities.

Despite these known patterns of community firearm violence concentration, and despite increasing acknowledgement and examination of violence in the US as a complex social and health issue with structural roots (29), the tendency to view violence largely as an individual-level problem remains in many sectors, including public health. This absence of a comprehensive structural analysis of interpersonal violence discounts the myriad ways that systems, policies, and practices have created the racialized conditions in which community violence flourishes alongside various other poor health and safety outcomes. In their paper “Racism and Structural Violence: Interconnected Threats to Health,” Sharif et al. detail the historical context and some contemporary examples of the interconnected, structural relationship between racism, violence, and health in the US (30). Dating back to European colonialism, racial capitalism—a capitalist economy which centers race in structuring social and labor hierarchies (31)—has dictated and championed the violent extraction and accumulation of capital through exploitation and commodification of marginalized racial groups. In utilizing the construct of race as a tool to leverage power, white supremacist principles have been entrenched in (and across) systems (e.g., education, housing, healthcare, immigration systems) by design. This historical foundation has also given way to a longstanding, inherited practice among those in positions of power (i.e., those in elevated positions of racial caste [i.e., white Americans] or those in elevated positions of social class [i.e., wealthy Americans]) of disassociating from violence and injustice in the name of optimization and efficiencies. This disassociation has been made all the easier by a lack of adequate language to unveil and challenge these normalized structures of violence.

While a racialized division of labor continues in present-day, with Black and Latinx individuals occupying disproportionately more low-pay, high-risk jobs (32), labor is not the only area that upholds the foundational power hierarchy. Other systemic inequities (e.g., in health, wealth, safety, income, education) associated with increased risk of community firearm violence exposure are also maintained along lines of race, class, gender, and disability, as the violence levied against marginalized groups persists in a more structural form. This manifestation of violence is particularly nefarious because it is an “avoidable impairment of fundamental human needs” (12); one which need not be, but persists owing to its deep embeddedness in institutions, systems, and structures of everyday life and which benefits those with the power and privilege to change it (33). The harm of structural violence is compounding and self-perpetuating, carrying forward the very white supremacist ideologies which beget it. Its structural nature makes it difficult to detect to an uncritical eye, thus it is widely conceived as “normal” (30).

As dominant narratives describing interpersonal firearm violence research and prevention continue to fail to adequately link the phenomenon of community violence with structural racism and other forms of structural violence, we continue to see the broad absolution of systems and structures in creating, maintaining, and exacerbating racialized patterns of interpersonal firearm violence in the US. This failure has also allowed our society to respond to the incredible grief and loss experienced by generations of minoritized communities largely with explicit and implicit indifference, along with a sense of inevitable persistence of that violence. The language we use to describe community gun violence, then, determines where power lies in shaping our understanding of its causes and solutions.

## From narratives of violence to healing and peacemaking

Recognizing and responding to community gun violence through a structural lens (i.e., as a deeply intertwined product of structural violence) requires narratives that go beyond calls for individuals to “stop shooting” or actions that rely predominantly on individual-level changes. The goal of such narrative change is not to simply offer new language or conceptualizations for communities impacted by violence to better cope or contextualize their own experiences; the narratives around community violence must shift among policy leaders, decision-makers, funders, media, and others who help shape responses to this issue. Parallel to the ways in which broadening and adopting a positively-oriented definition of health as “not merely the absence of disease” (34) has helped to embed “social determinants of health” in the mainstream of public health thinking and has fueled new investments, new lines of inquiry and research, and new frameworks for instituting practice and policy (35), we argue that substantial and long-lasting reductions in community violence require a similar reframing to counter status quo perspectives, alter power and resource allocations, and resist the continuance of policies and activities that promote or perpetuate health and safety inequity. Narrating the interrelationship between structural and community violence requires broad and positive (re-)conceptions of gun violence prevention and public safety as more than the mere absence of (or desistence from) violence, but also, and significantly, the existence and persistence of systems of support for health, healing, transformation, and peace(−making).

In Baltimore, Maryland, for example, a community-led movement involving the promotion of recurring, weekend-long ceasefires on gun violence recently rebranded itself to emphasize the power and necessity of a focus on collective peace-building. Coupled with the removal of an image of a gun from its logo, this evolution followed the incorporation of a proactive, year-round “peace challenge” where community members citywide are not only challenged to desist from violence during the weekend hours, but are also supported to organize regular peace-building activities—rallies, resource fairs, concerts, vigils, poetry readings—to help one another connect to community and resources to address root causes of violence (36). Leaders of the movement have described this rebranding effort as shifting the focus away from what they are against—violence—and instead bringing attention to what they are for:

*“We're not saying it's an anti-violence movement because that's what we're against. Everybody knows you're against violence but being against violence just creates more struggle with violence and focusing on a power of violence and that's not what we want for ourselves. So, we're only focusing on how we're a peace movement, a love movement, a joy movement.”*

*–* Erricka Bridgeford, co-organizer of Baltimore’s “Ceasefire” movement (37).

However, this shift in language is not just about replacing one word with another. By creating a new center of gravity—peace, rather than violence—Ms. Bridgeford and other “Baltimore Ceasefire 365” leaders are able to not only reclaim what “we want for ourselves,” but also redirect those committed in the movement, including investors, city agencies, and other promoters and supporters to reposition their relationship with the movement in ways that acknowledge this new gravitational center. Enacting a pro-healing, pro-peacebuilding (rather than solely or primarily an anti-violence) movement requires that communities have agency to self-determine what they need to thrive; in essence, they must have the power to self-determine what they need to counter structural violence and create healing-centered spaces that foster love, joy, and peace. Historical and present-day policies and practices have not just created and maintained structural inequities—intergenerational poverty, lack of access to economic opportunity, mass incarceration, and concentrated disinvestment in basic requisites of life such as food, housing, and schools—that increase risk of community firearm violence exposure and involvement; they have also intentionally excluded communities most impacted by structural violence from building and reshaping the communities in which they want to live. Thus, redressing the harms of structural violence means incorporating a framework of “situated multidimensional representation” (38) that equips those very communities with the authority and capacity to counter misrepresentation, reclaim valid narratives, and choose their own paths to safety through healing and peacebuilding.

The national movement to “Fund Peace” (39) was similarly a community-driven campaign to not only change narratives around the causes and consequences of recurring and persistent interpersonal gun violence in Black and Brown communities nationwide, but also, and most importantly, direct financial resources and political commitments towards community-led approaches to building peace. Started by the Black and Brown Peace Consortium (40), which began in 2018 as a coalition of advocates, researchers, policymakers, gun violence survivors, and practitioners dedicated to creating “sustainable pathways to opportunity, justice, and peace in our cities,” the “Fund Peace” campaign called for federal leaders to invest money and political will toward developing and expanding personnel, capacity, and infrastructure for violence intervention and prevention efforts led by those who have been working to build peace in their communities for years without consistent or adequate resources. In the weeks following President Joe Biden’s signing of an executive order to advance racial equity and support for underserved communities through the federal government (41), and amidst the disturbing surge in gun violence following the onset of the COVID-19 pandemic, members of the Black and Brown Peace Consortium met with his Administration to call for an alignment of values and action through a $5 billion federal investment toward building a comprehensive health and safety ecosystem, centering “homegrown peacemakers” as the cornerstones of this new approach to violence reduction. The Biden-Harris Administration responded to this call by not only committing $5 billion over 8 years towards community-based violence intervention in the Build Back Better Act, but by also expanding the criteria for existing grant programs, Medicaid reimbursement funds, and expenditures tied to COVID-19 relief monies to include the strategies backed by the “Fund Peace” campaign. These unprecedented investments in community-driven responses to gun violence, along with growing recognition and support of community experts to guide implementation and training of the responses, illustrate how shifts in narrative and language can also lead to shifts in the distribution of resources and power.

Narrative change and the redistribution of resources through the empowerment of those impacted by both community gun violence and structural violence must not only occur at the federal level. Some cities, for instance, have begun implementing the democratic practice of participatory budgeting, in which residents have direct say in deciding how to allocate government dollars in their respective locales. By empowering structurally marginalized taxpayers to help determine how their monies are spent, these jurisdictions are moving towards centering the voices and experiences of those who have the most to gain or lose by addressing or perpetuating structural violence. In this way, conventional “structure vs. agency” or “deficit vs. asset” dialectics can instead be used in tandem to create a more complete view of and into the complex personhood and experiences of those most affected by firearm violence, situating community-driven strategies such as healing and peacemaking as integral to our understanding of those experiences and the production of structurally rooted responses to community firearm violence.

## Conclusion

Centering the language of structural violence in the study and practice of community firearm violence prevention, while also elevating the perspectives and actions of healers and peacemakers to counter systems of oppression and create safety, is long overdue. To accurately understand and address the complex antecedents of community firearm violence in the US, a structural lens must be applied, making accessible inspection of those factors that are at the root of the root causes. While study of social determinants of interpersonal firearm violence has gained mainstream public health attention, discussion of structural violence, including structural racism and racial capitalism, is less common (33, 42). This omission has led to policies and practices that are insufficient, if not harmful, to communities most affected by this violence. It is critical that communities be empowered to conceptualize, and funded to execute, strategies to build peace and health in their spaces. Expanding interpersonal firearm violence narratives such that the goal of prevention and intervention efforts is not merely the absence of violence but rather the creation of a community safety and health ecosystem that recognizes and centers the humanity of those most impacted is essential to meet this critical moment in responding to community firearm violence—equitably and sustainably—for all communities.

## Data availability statement

The original contributions presented in the study are included in the article/supplementary material, further inquiries can be directed to the corresponding author.

## Author contributions

All authors listed have made a substantial, direct, and intellectual contribution to the work and approved it for publication.

## Conflict of interest

The authors declare that the research was conducted in the absence of any commercial or financial relationships that could be construed as a potential conflict of interest.

## Publisher’s note

All claims expressed in this article are solely those of the authors and do not necessarily represent those of their affiliated organizations, or those of the publisher, the editors and the reviewers. Any product that may be evaluated in this article, or claim that may be made by its manufacturer, is not guaranteed or endorsed by the publisher.
